# Hypothermia Associated With Impaired Consciousness in an Older Woman With Parkinson’s Disease: A Case Report

**DOI:** 10.7759/cureus.104225

**Published:** 2026-02-25

**Authors:** Shoji Kikui, Kenji Murakata, Daisuke Danno, Takao Takeshima

**Affiliations:** 1 Neurology, Tominaga Hospital, Osaka, JPN; 2 Headache Center, Department of Neurology, Tominaga Hospital, Osaka, JPN

**Keywords:** autonomic dysfunction, hypothermia, impaired consciousness, japanese geriatrics, parkinson’s disease

## Abstract

Parkinson’s disease (PD) is a progressive neurodegenerative disorder characterized by both motor and non-motor manifestations, including autonomic involvement. Severe thermoregulatory disturbances, such as hypothermia, are uncommon but clinically significant complications of PD. We report a case of hypothermia associated with impaired consciousness in an older woman with PD in the absence of overt cold exposure. A 78-year-old Japanese woman with PD was admitted with moderate-to-severe hypothermia and altered mental status despite living in a well-heated indoor environment during winter. Laboratory and imaging studies revealed no alternative causes of hypothermia. Supportive management with intravenous fluids and active external rewarming resulted in rapid clinical recovery, and no recurrence was observed during a two-year follow-up period. This case highlights hypothermia as a possible thermoregulatory complication in PD. Clinicians should be aware of this potentially reversible condition in patients with PD who present with acute neurological deterioration, even in the absence of obvious environmental risk factors.

## Introduction

Parkinson’s disease (PD) is a progressive neurodegenerative disorder characterized by motor symptoms and a wide spectrum of non-motor manifestations. Among these, autonomic dysfunction represents a key clinical feature and involves multiple systems, including the cardiovascular, gastrointestinal, urogenital, and thermoregulatory systems. Autonomic involvement is also a prominent feature in other neurodegenerative disorders, such as multiple system atrophy, underscoring its clinical relevance across Parkinsonian syndromes. Disturbances in thermoregulation have been described since the earliest clinical observations of PD and reflect the multisystem nature of the disease [1]. Severe thermoregulatory dysfunction may lead to acute clinical deterioration and therefore has significant diagnostic and management implications in patients with PD.

Thermoregulatory dysfunction in patients with PD commonly manifests as heat or cold intolerance, abnormal sweating, or altered peripheral temperature perception and is usually mild, primarily affecting quality of life. However, in rare cases, severe dysregulation may result in life-threatening conditions, such as hyperthermia or hypothermia [2]. Hypothermia associated with PD is uncommon and has been reported mainly in isolated case reports or small case series [3-5].

Accumulating evidence suggests that hypothermia in patients with PD cannot always be explained solely by environmental factors. In addition to isolated case reports, small case series and clinicopathological observations have described spontaneous hypothermia in patients with PD without clear external triggers [3-5]. Renga et al. reported a patient with PD who developed spontaneous periodic hypothermia and demonstrated Lewy body pathology involving the hypothalamus, indicating a potential role of this brain region in central thermoregulatory failure in PD [5]. These findings suggest that hypothermia may reflect thermoregulatory vulnerability related to central autonomic involvement in patients with PD. Clinically, failure to recognize hypothermia may lead to the misattribution of acute neurological deterioration to infection, medication effects, or disease progression, potentially delaying appropriate management. Despite these observations, hypothermia remains under-recognized in clinical practice, and its clinical characteristics and presentation in patients with PD have not been fully characterized.

Here, we report a case of hypothermia accompanied by impaired consciousness in an older woman with PD, occurring in the absence of apparent cold exposure, and discuss its clinical implications.

## Case presentation

A 78-year-old Japanese woman was admitted to the high-care unit of our hospital with hypothermia and impaired consciousness. Although the event occurred during February, one of the coldest months in Japan, the patient had been living indoors in a well-heated environment and had no history of cold exposure. She had a medical history of angina pectoris, for which her general practitioner prescribed aspirin (100 mg/day) and a transdermal nitroglycerin patch (27 mg/day).

**Figure 1:**
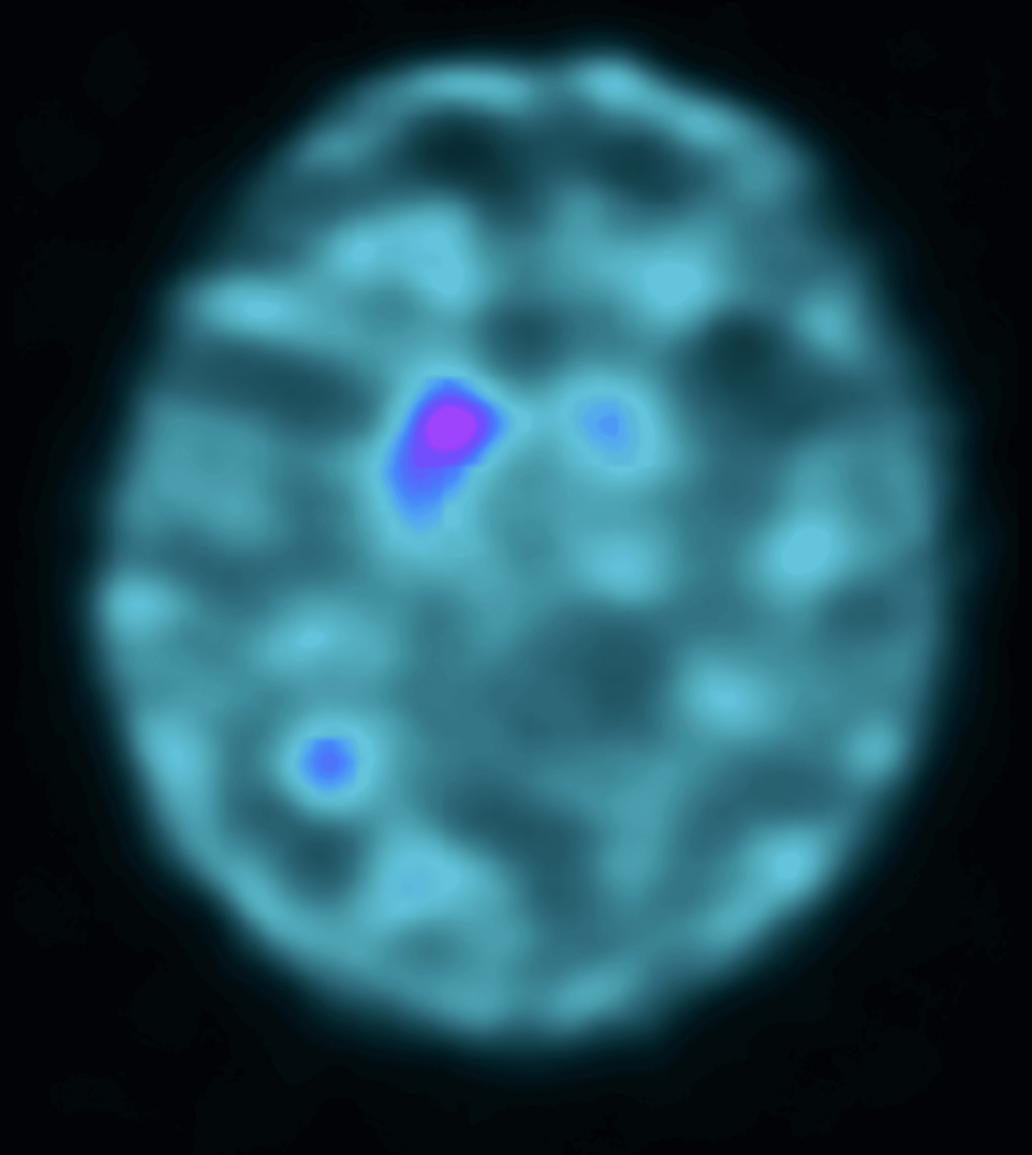
Dopamine transporter single-photon emission computed tomography (DAT-SPECT). DAT-SPECT demonstrated markedly reduced striatal uptake with specific binding ratios of 1.49 on the right and 0.91 on the left. The asymmetric reduction of tracer uptake provides objective evidence of presynaptic dopaminergic degeneration and supports the diagnosis of Parkinson’s disease.

Two years before admission, the patient began to experience bradykinesia and a shuffling gait. She consulted her general practitioner at that time, and because she also complained of bilateral hip pain, she was diagnosed with bilateral hip osteoarthritis and managed conservatively with medication and rehabilitation. Despite treatment, her gait disturbance did not improve and gradually progressed. Approximately six months before presentation to our department, she developed a resting tremor in her right hand. The progressive nature of her gait disturbance, together with the emergence of unilateral resting tremor, raised suspicion of Parkinsonism, and she was referred to our hospital for neurological evaluation. Neurological examination revealed typical asymmetric Parkinsonian motor features, including masked facies, right-dominant rigidity, resting and postural tremor, stooped posture, shuffling gait, and postural instability. Constipation and nocturia were present as non-motor features suggestive of mild autonomic involvement. However, no clinically significant orthostatic hypotension, severe sudomotor dysfunction, or other objective evidence of generalized autonomic failure was observed. Brain magnetic resonance imaging revealed mild cerebral atrophy and periventricular ischemic changes without any acute lesions. Magnetic resonance angiography revealed an occlusion at the origin of the right middle cerebral artery. After consultation with the stroke neurology service, this finding was considered asymptomatic, and continuation of aspirin therapy was recommended. Dopamine transporter single-photon emission computed tomography revealed markedly decreased specific binding ratios, with values of 1.49 on the right and 0.91 on the left (Figure 1).

Based on these clinical features and imaging findings, the patient was diagnosed with PD, corresponding to Hoehn and Yahr stage III [6]. No red flags suggestive of atypical Parkinsonism were observed, including early severe autonomic failure, cerebellar signs, pyramidal signs, rapid progression, or early unexplained falls during the first year after symptom onset. Treatment with a levodopa/carbidopa combination was initiated at a dose of 100 mg/day and subsequently increased to 200 mg/day, which resulted in clear clinical improvement of resting tremor and gait disturbance.

Upon admission, her parameters were as follows: height, 155 cm; body weight, 60 kg; rectal temperature, 31.6 °C; blood pressure, 131/63 mmHg; and pulse rate, 63 beats/min with a regular rhythm. Cardiac and respiratory examinations revealed no remarkable abnormalities, and her peripheral oxygen saturation was 97%.

Her level of consciousness was mildly impaired, with a Glasgow Coma Scale score of 14 (E4, V4, and M6) points [7]. Bilateral pupillary light reflexes were diminished. Neurological examination revealed moderate muscle rigidity, predominantly on the right side, without any tremors.

The laboratory findings are summarized in Table 1. They revealed mild anemia, thrombocytopenia, and transient elevation of liver enzymes. Additionally, thyroid-stimulating hormone levels were mildly elevated. Conversely, free thyroxine levels were within the normal range. Moreover, parameters were normal for renal function, electrolyte levels, coagulation parameters, and serum levels of vitamin B1, vitamin B12, and folate. C-reactive protein levels were also not elevated, indicating no significant inflammatory response.

**Table 1:** Laboratory findings at admission and at discharge. Transient abnormalities in liver function tests observed at admission normalized by discharge. No significant inflammatory response or metabolic derangements were detected. RBC: red blood cell count, Ht: hematocrit, WBC: white blood cell count, PLT: platelet count, AST: aspartate aminotransferase, ALT: alanine aminotransferase, LDH: lactate dehydrogenase, γ-GT: gamma-glutamyl transferase, BUN: blood urea nitrogen, CRE: creatinine, GLU: glucose, CK: creatine kinase, Na: sodium, K: potassium, Cl: chloride, CRP: C-reactive protein, PT: prothrombin time, APTT: activated partial thromboplastin time, TSH: thyroid-stimulating hormone, fT4: free thyroxine

Parameter	At admission	At discharge	Reference range
RBC (×10^4^/μL)	380	421	380-480
Ht (%)	33.1	36.7	35.0-47.0
WBC (/μL)	6,690	5,360	4,000-8,000
PLT (×10^4^/μL)	14.6	28.5	14.0-35.0
AST (IU/L)	291	16	10-40
ALT (IU/L)	468	29	5-40
LDH (IU/L)	331	262	115-245
γ-GT (IU/L)	66	34	0-30
BUN (mg/dL)	23.3	15.5	6.0-20.0
CRE (mg/dL)	0.5	0.6	0.4-0.8
GLU (mg/dL)	108	99	70-110
CK (U/L)	124	108	32-180
Na (mEq/L)	142	142	137-148
K (mEq/L)	5.3	4.0	3.6-5.0
Cl (mEq/L)	103	104	98-109
CRP (mg/dL)	0.20	0.20	<0.30
PT (sec)	12.3	-	10.5-13.5
APTT (sec)	32.4	-	25.0-40.0
D-dimer (μU/mL)	0.5	-	0.0-1.0
TSH (μU/mL)	5.50	-	0.54-4.54
fT4 (ng/dL)	1.66	-	0.97-1.72
Vitamin B1 (ng/mL)	28	-	24-66
Vitamin B12 (pg/mL)	833	-	233-914
Folate (ng/mL)	6.4	-	2.4-9.8

**Figure 2:**
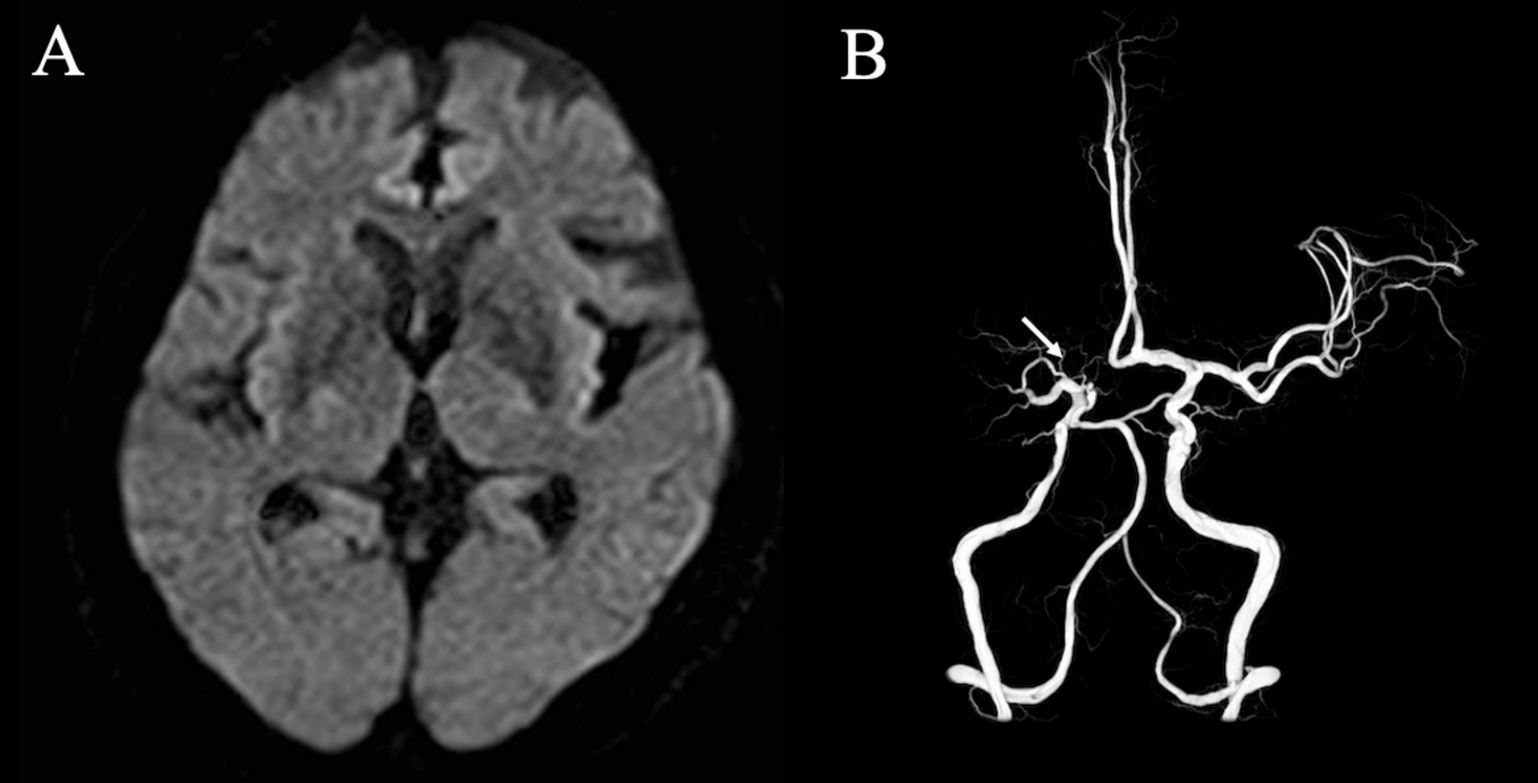
Neuroimaging findings at admission. (A) Diffusion-weighted imaging showed no evidence of acute ischemic lesions. (B) Magnetic resonance angiography demonstrated chronic occlusion at the origin of the right middle cerebral artery (M1 segment) arising from the internal carotid artery bifurcation (arrow).

Electrocardiography showed no Osborn waves, and chest radiography showed no abnormalities. Brain magnetic resonance imaging revealed no acute or new structural abnormalities compared with those observed on previous imaging (Figure 2A). Magnetic resonance angiography showed no interval change, including persistent occlusion at the origin of the right middle cerebral artery (Figure 2B).

After admission, intravenous fluid therapy and active external rewarming with a thermal blanket were initiated. No adjustment of levodopa/carbidopa was made during the hypothermic episode. She was successfully rewarmed within six hours, and hypothermia did not recur after discontinuation of warming measures. On the following day, her level of consciousness improved, and she was allowed oral intake. By hospitalization day 3, her level of consciousness had improved to a Glasgow Coma Scale score of 15 (E4, V5, M6), and the patient was transferred from the high-care unit to the general ward. Active rehabilitation was initiated after transfer. The dosage of levodopa was increased to 300 mg/day due to worsening muscle rigidity. The transient liver dysfunction observed at admission resolved spontaneously without specific treatment. By hospitalization day 16, her activities of daily living had recovered to nearly the same level as before admission. She was fully conscious when discharged and scheduled for outpatient follow-up. During a two-year follow-up period after discharge, the patient remained clinically stable without further episodes of hypothermia.

## Discussion

In the present case, an older woman with PD developed moderate-to-severe hypothermia accompanied by impaired consciousness despite the absence of apparent cold exposure in a heated indoor environment. Hypothermia resolved rapidly with supportive rewarming alone, without modification of antiparkinsonian therapy, and did not recur during a two-year follow-up period. This favorable and non-recurrent course suggests that the episode may have reflected transient thermoregulatory instability rather than established or progressive autonomic failure.

Previous reports have described several characteristic features of hypothermia in patients with PD, including winter onset, impaired consciousness at presentation, and transient worsening of Parkinsonian motor symptoms, such as rigidity and bradykinesia [3-5]. As reported by Kataoka et al. [4], hypothermic episodes in patients with PD often improve within hours to days following supportive rewarming and do not necessarily recur. The present patient exhibited a similar clinical course, suggesting that hypothermia in patients with PD may represent acute decompensation in susceptible individuals rather than persistent autonomic collapse.

Thermoregulation depends on a complex network involving the hypothalamus, brainstem, spinal autonomic pathways, and peripheral autonomic nerves. In PD, α-synuclein pathology extends beyond the nigrostriatal system and involves central and peripheral autonomic structures. Lewy body pathology in the hypothalamus has been described in patients with PD who developed spontaneous hypothermia, supporting the possibility of central thermoregulatory vulnerability [5]. Rather than indicating generalized autonomic failure, such central involvement may lower the threshold for temperature dysregulation under certain conditions.

In the present case, the objective evidence of generalized autonomic failure was limited. Apart from constipation and nocturia, no marked orthostatic hypotension, severe sudomotor dysfunction, or other major autonomic abnormalities were documented. The absence of recurrent episodes over two years further argues against ongoing or severe autonomic collapse. These findings suggest that Parkinsonism may have acted as a predisposing factor conferring thermoregulatory vulnerability, rather than serving as a sole causal mechanism.

Environmental or behavioral factors may also contribute. Epidemiological studies in Japan have demonstrated an increased risk of winter hypothermia among older adults and patients with neurological disorders, including PD, a phenomenon sometimes referred to as “urban-type hypothermia” [4]. Even in heated indoor environments, seasonal temperature fluctuations, reduced mobility, or impaired behavioral thermoregulation may act as triggers. In this context, PD-related vulnerability may interact with external factors to precipitate hypothermia.

Electrocardiographic Osborn waves, which have been described in severe cases [1], were not observed in this patient, indicating that clinically significant hypothermia in PD can occur without typical electrocardiographic findings. Transient laboratory abnormalities, including liver dysfunction, were most likely secondary to systemic hypothermic stress rather than primary organ pathology.

Clinically, hypothermia in patients with PD appears to be highly reversible when promptly recognized and appropriately managed. Awareness of the potential thermoregulatory vulnerability in patients with PD, particularly during the winter months, may facilitate early recognition and prevent diagnostic delay. Rather than concluding that hypothermia represents established autonomic failure, this case highlights the importance of considering temperature dysregulation as a possible but uncommon complication in patients with PD.

This report has several limitations. As a single-case study, causality between PD and hypothermia cannot be definitively established. Objective autonomic testing was not systematically performed, and therefore, subclinical autonomic dysfunction cannot be excluded. In addition, environmental or behavioral factors may have contributed to the episode. Further accumulation of similar cases and systematic investigation are required to better clarify the mechanisms and clinical significance of hypothermia in patients with PD.

## Conclusions

Hypothermia in patients with PD is an uncommon but clinically important complication that may present with acute disturbance of consciousness or sudden motor deterioration. This case illustrates that hypothermia can occur even in a heated indoor environment and in the absence of overt cold exposure, highlighting possible thermoregulatory vulnerability in PD. Because hypothermia is potentially reversible when promptly recognized and appropriately managed, it should be considered in patients with PD who present with acute neurological deterioration, particularly during winter.
